# Variable Anterior Segment Dysgenesis and Cardiac Anomalies Caused by a Novel Truncating Variant of FOXC1

**DOI:** 10.3390/genes13030411

**Published:** 2022-02-24

**Authors:** Mariya R. Ahmed, Saumil Sethna, Laura A. Krueger, Michael B. Yang, Robert B. Hufnagel

**Affiliations:** 1 Medical Genetics and Ophthalmic Genomics Unit, National Eye Institute, National Institutes of Health, Bethesda, MD 20892, USA; mariya.ahmed@nih.gov; 2 Department of Otorhinolaryngology—Head & Neck Surgery, University of Maryland School of Medicine, Baltimore, MD 21201, USA; ssethna@its.jnj.com; 3 Department of Ophthalmology, Division of Pediatric Ophthalmology, Abrahamson Pediatric Eye Institute, University of Cincinnati College of Medicine, Cincinnati Children’s Hospital Medical Center, Cincinnati, OH 45229, USA; laura.krueger@uky.edu (L.A.K.); michael.yang@cchmc.org (M.B.Y.)

**Keywords:** *FOXC1*, in vitro studies, novel variant, ophthalmic genetics, intrafamilial variability, anterior segment dysgenesis

## Abstract

Anterior segment dysgenesis (ASD) encompasses a wide spectrum of developmental abnormalities of the anterior ocular segment, including congenital cataract, iris hypoplasia, aniridia, iridocorneal synechiae, as well as Peters, Axenfeld, and Rieger anomalies. Here, we report a large five-generation Caucasian family exhibiting atypical syndromic ASD segregating with a novel truncating variant of *FOXC1*. The family history is consistent with highly variable autosomal dominant symptoms including isolated glaucoma, iris hypoplasia, aniridia, cataract, hypothyroidism, and congenital heart anomalies. Whole-exome sequencing revealed a novel variant [c.313_314insA; p.(Tyr105*)] in *FOXC1* that disrupts the α-helical region of the DNA-binding forkhead box domain. In vitro studies using a heterologous cell system revealed aberrant cytoplasmic localization of FOXC1 harboring the Tyr105* variant, likely precluding downstream transcription function. Meta-analysis of the literature highlighted the intrafamilial variability related to FOXC1 truncating alleles. This study highlights the clinical variability in ASD and signifies the importance of combining both clinical and molecular analysis approaches to establish a complete diagnosis.

## 1. Introduction

Anterior segment dysgenesis (ASD) encompasses a wide spectrum of developmental abnormalities of the anterior ocular segment coupled with increased risk of glaucoma, Peters anomaly, iris hypoplasia, corectopia, or sclerocornea, which may lead to blindness [1]. Syndromic ASD is caused by specific genes, predominantly transcription factors and cofactors such as *PAX6*, *PITX2*, and *FOXC1* [2,3,4,5,6,7,8], that are critical for the development of many tissues, including ocular, neuronal, craniofacial, dental, cardiac, and renal. Clinical heterogeneity, both phenotypic and genotypic, along with the overlapping of symptoms associated with anterior segment dysgenesis make the disease classification and clinical diagnosis challenging. This has been the case despite several attempts to categorize ASD to aid the diagnosis [9,10,11].

Forkhead box C1 (*FOXC1*) belongs to a family of transcription factor genes, and heterozygous variants have been associated with Axenfeld-Rieger syndrome and ASD [12,13,14]. FOXC1 plays a critical role during early development, specifically for the development of the anterior segment of the eye, including iris, lens, and cornea. Further, FOXC1 is involved in the normal development of the kidney, brain, and heart [15,16,17]. More than 50 different variants in *FOXC1* have been associated with Axenfeld-Rieger syndrome type 3. Variants may reduce the amount of functional FOXC1 that is produced or affect the DNA binding capability of FOXC1, resulting in functional haploinsufficiency, leading to variable ASD in humans and mice [18,19]. Variable expressivity associated with FOXC1 variants may be quite broad, with the observation of Peters anomaly, iris hypoplasia, or corectopia among family members harboring the same causal variant [13]. Here, we describe a novel truncating variant in FOXC1 causing autosomal dominant atypical ASD with highly variable phenotypic spectrum including variable iris hypoplasia and aniridia in a large Caucasian family. We also perform an extensive meta-analysis of the literature to explore the range and frequency of phenotypes related to FOXC1 truncating alleles segregating in families.

## 2. Materials and Methods

### 2.1. Clinical Evaluation

This study was conducted under institutional review board-approved protocols in accordance with the Declaration of Helsinki for the release of clinical information, family history, blood draw, and skin biopsies. Informed consent was obtained after the explanation of the study’s risks and benefits.

### 2.2. Whole Exome Sequencing (WES) and Sanger Sequencing

WES and data analysis were performed as described previously [20]. Exome sequencing was conducted on two affected individuals and was enriched using the Nimblegen SeqCap EZ Exome v2.0 Library (Roche Diagnostics; San Francisco, CA, USA). Segregation analysis of the *FOXC1* variant was performed using Sanger sequencing on the DNA samples of the seven participating family members.

### 2.3. In Vitro Localization Analysis

We subcloned human *FOXC1*, a single-coding exon gene, from genomic DNA samples, into tdTomato-C1 vector and mutant FOXC1 into GFP-C2 (Clonetech) with Bgl II and Sal I sites using infusion technology. Constructs were validated using Sanger sequencing. Seventy percent confluent COS-7 cells were transfected using lipofectamine 2000. The medium was replaced with 2% FBS-DMEM and then 24 h later it was replaced with 4% FBS-DMEM. After another 24 h cells were fixed with 4% PFA for 15 min at room temperature, stained with DAPI to visualize nuclei and Phalloidin-647 to visualize the actin cytoarchitecture in PBS with 0.1% triton-X to permeabilize the cells, washed 3 times with PBS, and mounted on slides. The cells were imaged using a Zeiss 710 confocal microscope with 0.5 µm step size and processed using ImageJ as detailed previously [21].

### 2.4. Meta-Analysis

A literature review of intrafamilial variability in FOXC1-related disorders due to truncating variants was performed using The Human Gene Mutation Database (http://www.hgmd.cf.ac.uk/ac/index.php; last accessed on 18 January 2022). Initially, a comprehensive review of 134 publicly available reports of *FOXC1*-related disorder variants was performed to identify those with truncating variants (*n* = 41). Further narrowing was performed to yield a final meta-analysis that reported detailed phenotypes of multiple affected family members. These final variants were comprehensively compiled and analyzed.

## 3. Results

### 3.1. Patient Characteristics

**Figure 1:**
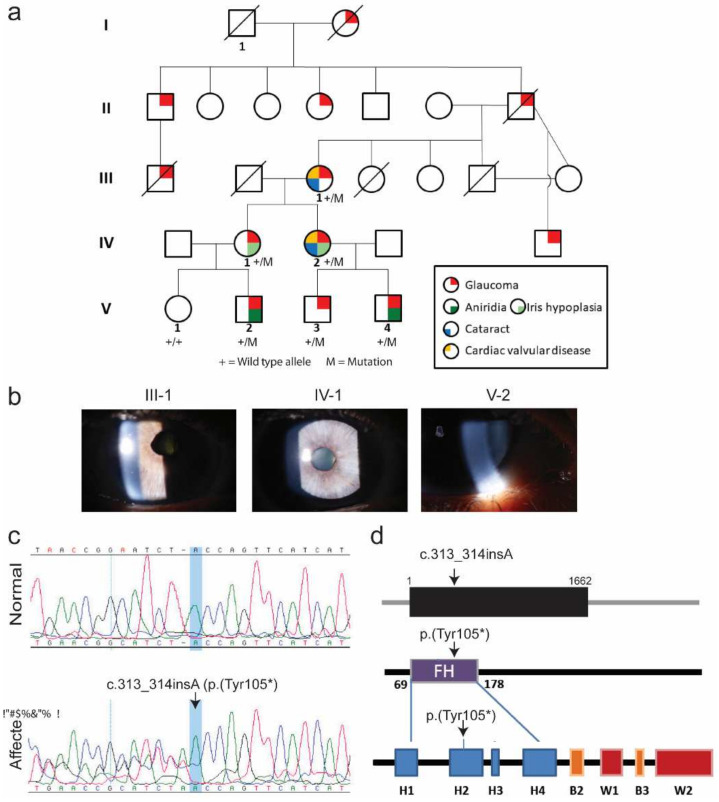
(**a**) Numbered individuals received a dilated eye examination. The predominant phenotype was congenital glaucoma, while other ocular and nonocular abnormalities exhibited variable expression; (**b**) slit lamp analysis revealed multiple eye problems. The proband (IV-1) is a 38-year-old female with congenital glaucoma, bilateral iris hypoplasia, and hypothyroidism. Family history is consistent with a highly variable autosomal dominant condition that includes isolated glaucoma (III-01), iris hypoplasia (IV-01), aniridia (V-02), cataract, hypothyroidism, and congenital heart anomalies; (**c**) representative Sanger sequencing chromatograms of *FOXC1* exon from a normal (V-1) and an affected (V-2) individuals of family LNG5, showing the c.313_314insA allele. All individuals included within the LNG5 pedigree were tested with Sanger sequencing to confirm segregation and full phenotypic penetrance; (**d**) schematic representation of FOXC1 gene and protein structure. Two nuclear localization signals (NLS1 and NLS2) are present at both ends of the DNA-binding forkhead box domain (FH). *FOXC1* c.313_314insA [p.(Tyr105*)] disrupts the α-helical region of the FH domain and removes NLS2 along with the carboxy tail. H—Helix. B—β-sheets. W—Winged helix.

The proband was a 38-year-old Caucasian female who presented with congenital glaucoma, bilateral iris hypoplasia, and hypothyroidism. She is part of the fourth generation of a five-generation family with a history of vision impairment along with multi-organ disorder. There were 12 affected individuals documented across five generations in this family, with glaucoma being the most predominant segregating phenotype. Out of the affected family members, seven were male and five were female. Numbered individuals (Figure 1a) in the family, across a range of ages (21–68 years), received a dilated eye exam as part of a comprehensive evaluation of visual function, and, based on clinical history, evaluation of cardiac and renal function.

### 3.2. Clinical Examination

Clinical data and blood samples for DNA extraction were obtained from seven family members with consent and made available for this study. The family history is consistent with an autosomal dominant inheritance pattern presenting with highly variable symptoms including isolated glaucoma (12 out of the 25 family members), iris hypoplasia, aniridia, cataract, hypothyroidism, and congenital heart anomalies including mitral valve prolapse. (Figure 1a,b). Other individuals, such as the proband and the proband’s mother (IV-1 and III-1, respectively), had a variety of phenotypes: ranging from cardiac valvular disease to iris hypoplasia to glaucoma.

### 3.3. Genetic Analysis

Whole exome sequencing was performed on two individuals, revealing a novel insertion variant (c.313_314insA) in *FOXC1* (Figure 1c). This variant was predicted to lead to early protein truncation [p.(Tyr105*)] (Figure 1c,d). Additionally, the variant is predicted to disrupt the α-helical region of the DNA-binding forkhead box domain. Segregation of the variant along with disease phenotype was then found in four additional family members (Figure 1b) and was absent from an unaffected family member (Figure 1c). The allele frequency of the truncating variant (c.313_314insA) was zero among the 126,000+ exomes reported in the gnomAD database. The highly variable phenotypes found in the assessed family spanned the entire spectrum of *FOXC1*-associated diseases and ranged from congenital glaucoma to Axenfeld-Rieger Syndrome (ARS). Axenfeld-Rieger Syndrome type 3 is an autosomal dominant condition primarily characterized by developmental abnormalities of the anterior segment and is also associated with systemic abnormalities such as cardiac defects and hearing loss (OMIM ID: 602482). Our plausible explanation of the observed variability of the phenotype, especially aniridia, observed in our family as compared to other known *FOXC1* alleles, might be the co-inheritance of a pathogenic variant of other ASD-associated genes along with the c.313_314insA allele. However, through exome analysis, we found no pathogenic variants in other ASD-associated genes, which likely excludes this possibility.

**Figure 2:**
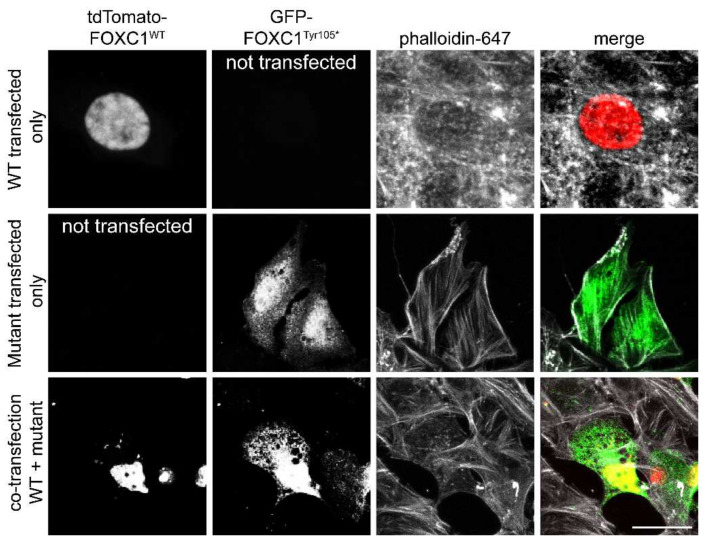
Representative images of COS-7 cells transiently transfected with tomato-tagged wild-type (WT) or FOXC1 constructs. As compared to WT-FOXC1, which is primarily localized in the nucleus, the truncated FOXC1 was also found in the cytoplasm. Scale bar—10 µm.

Next, to assess the functional impact of the novel *FOXC1* variant, we generated fluorescently tagged constructs, tdTomato-FOXC1^WT^, and GFP-FOXC1^p.Tyr105*^. Transient transfection in COS-7 cells showed that FOXC1^WT^ localized exclusively in the nucleus as expected. The p.Tyr105* is positioned in between two known nuclear localization sequences, NLS1 and NLS2. Hence, the variant-mediated early truncation was predicted to eliminate NLS2. Corresponding to the in silico prediction, overexpression of FOXC1^Tyr105*^ in COS-7 cells revealed that the variant protein was partially localized in the cytoplasm (Figure 2), as opposed to the exclusive nuclear localization of WT protein. The mislocalization of the FOXC1^Tyr105*^ protein may likely disrupt its downstream transcriptional function. Overall, our in vitro studies further support previous research that shows that both NLS1 and NLS2 motifs are essential for FOXC1 nuclear localization and without them, FOXC1 protein localizes improperly to cytoplasmic regions [22].

### 3.4. Literature Meta-Analysis

To further explore *FOXC1*-related intrafamilial variability, we performed a comprehensive literature review of early truncation alleles of *FOXC1* that segregated in multiple affected family members with available reported clinical phenotypes (Table 1; *n* = 12 publication, 56 affected individuals). A majority of the segregating variants were found across multiple generations (11/12). Similar to our study, we found extensive variability among intrafamilial phenotypes due to truncating variants (Table 1). All of the families had individuals who could be categorized as “more severe” due to increased severity and breadth of disease presentation, and individuals that could be categorized as “less severe” due to the isolated presentation of phenotypes. The variation in the range of intrafamilial disease severity is a general phenomenon among genetically inherited diseases, particularly prominent in ocular and systemic diseases. Overall, only one family had affected individuals with only nonsyndromic presentations (normal systemic features), five families with affected individuals who all had syndromic presentations, and six families with affected individuals that had either syndromic, and nonsyndromic presentations. This supports the fact that ASD cannot be ruled out in cases with nonsyndromic presentations and further clinical evaluation is recommended.

**Figure 3:**
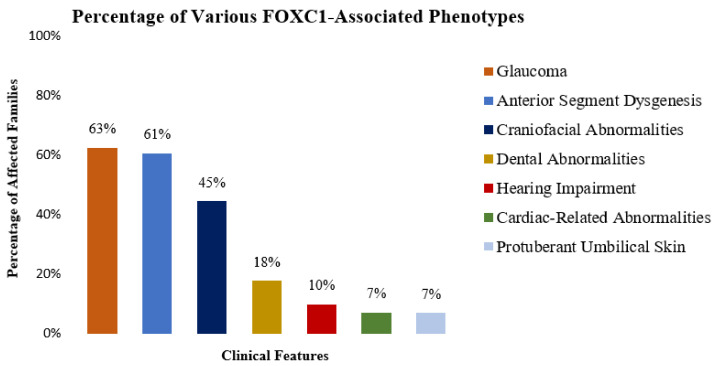
Prevalence of the commonly occurring clinical features among 56 individuals from 12 families affected with truncating variants of *FOXC1* from Table 1 (*n* = 56 individuals).

While Axenfeld-Rieger anomaly was listed as a key clinical diagnosis for 9 out of 12 of the *FOXC1* variants that are listed in Table 1, it does not capture the range of ocular phenotypes that were observed in these families. Sixty-three percent of affected individuals from Table 1 were affected with glaucoma and 61% were affected with ASD (Figure 3). Additionally, the range of ocular phenotypes include optic atrophy, Haab striae, iridocorneal adhesion, iris atrophy, corectopia, posterior embryotoxon, elevated intraocular pressure, aniridia, displaced Schwalbe line, corneal haze, phthisis bulbi, thickened cornea, iris heterochromia, corneal perforation, lens extrusion, retinal detachment, hyperplastic primary vitreous, abnormalities of the Bowman layer, abnormalities of the Descemet membrane, abnormalities of the corneal endothelium, corneal cysts, and megalocornea.

Further, differences in the frequency of FOXC1-associated phenotypes were noted. While relatively commonly occurring phenotypes are depicted in Figure 3, a significant number of features were not common and some features were only unique to singular families. Surprisingly, aniridia was not reported to be associated with *FOXC1* in any of the reviewed publications and to the best of our knowledge is a unique feature of the SNP associated with the *FOXC1-*affected family reported in this study. Moreover, common systemic abnormalities such as hearing impairment and cardiac-related abnormalities were less common, only seen in 10% and 7% of the studied affected individuals found in the meta-analysis, respectively.

## 4. Discussion

Here, we performed a genetic analysis on seven individuals from a large (12 of 25 family members were affected), multigenerational family, and we found a novel nonsense variant in *FOXC1* causing extensive variable phenotypic expressivity among family members, including variable iris hypoplasia and aniridia. Aniridia is a very uncommon *FOXC1-*related phenotype. We observed intrafamilial clinical heterogeneity, the proband’s sister (IV-2) presented with severe syndromic presentation including glaucoma, iris hypoplasia, cataract, and cardiac valvular disease, while other family members with the same variant had milder phenotypic presentations. IV-1 (proband) only presented with iris hypoplasia and glaucoma.

Meta-analysis of early truncation alleles of *FOXC1*-associated phenotypes revealed extensive intrafamilial and interfamilial heterogeneity of ocular and systemic phenotypes in 12 families. Variants in the *PAX6* gene account for >90% of all aniridia cases, regardless of whether they are familial (~66%) or sporadic (~33%) [32]. Previously, only one variant in *FOXC1* (c.454T>G; p.Trp152Gly) has been associated with aniridia [33]. We postulate that *FOXC1*-related aniridia appears to be the extreme end of iris hypoplasia rather than true aplasia as is seen in *PAX6*-related aniridia [34]. However, due to logistical limitations, we are not able to provide additional cellular validation to gain further insight into the real relevance of the variant. Future studies may include such proposed assays.

Among the variants present in the final meta-analysis, one other variant shared a common domain (helix 2) with the novel variant presented in this study. The family associated with c.317delA suffered from a variety of ocular abnormalities such as corectopia and iridocorneal adhesion and some of those phenotypes overlapped with those found in our reported family: glaucoma and iris hypoplasia. In terms of systemic phenotypes, our family was found to have abnormalities of the ocular, renal, and cardiac systems, whereas the c.317delA-associated family’s systemic phenotypes only reportedly extended to craniofacial abnormalities such as a flat broad nasal bridge or ocular hypertelorism [25]. In conclusion, the extent of similarities between the two families does not suggest a strong correlation based upon the shared protein domain.

Additionally, there were six other pathogenic variants found to affect the same NLS signal (NLS2) as the novel variant found in this study [6,14,25,26,27,28]. Among these six families, there was one family (c.367C>T-associated) that was reported to only have ocular phenotypes (non-systemic); however, this could be a limitation of clinical reporting. Besides this, the rest of the families all reported systemic phenotypes in addition to ocular phenotypes. The phenotypes affecting the other families ranged from atrial septal defect to short stature to dental abnormalities. Glaucoma was present/reported in five out of the six families and was the most commonly occurring phenotype among this subset [6,14,25,26,27]. Cardiac anomalies were present in one other family in this subset besides ours [26]. Overall, there was no clear correlation in phenotype among these six families that distinguished them from the other families reported in Table 1. In previous research, a strong genotype–phenotype correlation in *FOXC1* has not been able to be elucidated either, but it has been suggested that *FOXC1* duplications and mutations that disrupt the inhibitory domain have increased similarity in phenotype due to their similar mechanisms when compared to the phenotypes associated with FOXC1 missense variants that are associated with haplodeficiency [35,36,37].

The phenomes from the meta-analysis present a variety of ocular abnormalities ranging from Haab’s striae to cataract to microphthalmia with pronounced intrafamilial variability. In reference [31], the proband exhibited posterior embryotoxon, corectopia, megalocornea, and ocular hypertension but the affected sibling was only reported to have isolated glaucoma. Additionally, one proband was affected with a range of ocular abnormalities: glaucoma, microphthalmia, iris anomalies, and pupil anomalies, while the assessed parent presented with only glaucoma [31]. Often, the phenotypic presentation of the proband was the most severe among family members, which is not surprising since the proband prompted the genetic investigation.

A review of the meta-analysis revealed additional variability among systemic features related to *FOXC1.* While the predominant abnormalities were ocular, *FOXC1-*related abnormalities also extended into other organ systems. Three families reported having afflictions related to the cardiac system including the family reported in this study [24,27]. There were three families reported to have hearing impairments [24,27,30]. Interestingly, protuberant umbilical skin, considered a salient feature for FOXC1-related ARS, was only noted in two families. Global developmental delay was noted in only one family. However, not all of these phenotypes have a causally established association with a *FOXC1* variant. While we cannot exclude the possibility that certain phenotypes are due to secondary causes, such as a congenital malformation or a second Mendelian disease, the recurrent observations of systemic findings in similar tissues systems warrants consideration of systemic evaluations in all family members with *FOXC1*-related disorders.

The meta-analysis portion of our study further highlights the broad spectrum of phenotypes associated with *FOXC1*, both in the variability of tissues affected and their clinical co-morbidities. Surprisingly, the presentations that extended past ophthalmological afflictions into other organ systems were less common than expected. This is possibly due to incomplete inclusion of all clinical details or differences in systemic investigations performed in different reports across time, which is a limitation of a literature-based meta-analyses such as this. As such, the list of phenotypes described in Table 1 and Figure 3 may be underrepresented. However, our findings do recommend comprehensive ocular and systemic examinations, even when other family members are determined to have partial phenotypic expression of *FOXC1*-related disorders.

Despite the extensive phenotypic variability observed in individuals harboring *FOXC1* variants, the underlying mechanism of variability is currently unknown. Potential environmental factors/variability (e.g., drug exposure), reduced penetrance at the tissue level, subclinical phenotypic features, and/or genetic modifiers may partially explain the clinical variability. Future studies with a larger sample size would be required to test these rigorously.

In summary, our study further affirms the variability among individuals and families with *FOXC1*-related ARS, as well as diagnostic and testing challenges. ASD should be considered in apparently isolated congenital glaucoma or cataracts. The additional risk of congenital cardiac and renal disease should also be taken into consideration where ASD is noted. Therefore, the combination of systemic clinical and molecular analysis is likely the most efficient approach to establish a diagnosis and complete a systemic evaluation of ASD.

## Figures and Tables

**Table 1:** Compilation of the genetic and phenotypic information related to truncating variants associated with *FOXC1* segregating within multiple family members.

DNA Change	Amino Acid Change	Classification	Proband Phenotype	Intrafamilial Phenotypes	Reference
c.15delC	p.(Arg4Hisfs*43)	Frameshift	Congenital glaucoma, optic atrophy	2 member, 2 generations; optic atrophy, unilateral superior oblique palsy	[23]
c.67C>T	p.(Gln23*)	Nonsense	Posterior embryotoxon, iris hypoplasia, ocular hypertelorism, flat midface, microdontia, protuberant umbilical skin	9 members, 3 generations; posterior embryotoxon, iris hypoplasia, glaucoma, flat midface, microdontia, ocular hypertelorism, protuberant umbilical skin, hearing loss, cardiac defect	[24]
c.317delA	p.(Gln106Argfs*75)	Frameshift	Glaucoma, iridocorneal adhesion, iris atrophy, Haab’s striae, iris hypoplasia, ocular hypertelorism, telecanthus, flat face, flat broad nasal bridge	4 members, 2 generations; corectopia, posterior embryotoxon, elevated IOP, glaucoma, iridocorneal adhesion, iris hypoplasia, iris atrophy, flat midface, flat broad nasal bridge, normal intelligence	[25]
c.313_314insA	p.(Tyr105*)	Frameshift	Congenital glaucoma, bilateral iris hypoplasia, hypothyroidism	12 members, 5 generations; glaucoma, iris hypoplasia, aniridia, cataract, hypothyroidism, congenital heart anomalies (mitral valve prolapse)	Present Study
c.358C>T	p.(Gln120*)	Nonsense	Displaced Schwalbe line, iridocorneal adhesion, iris hypoplasia, glaucoma, leukoma, maxillary hypoplasia, ureteral stenosis	7 members, 3 generations; posterior embryotoxon, iridocorneal adhesion, iris hypoplasia, corectopia, glaucoma, maxillary hypoplasia, ocular hypertelorism, atrial septal defect, ureteral stenosis, protuberant umbilical skin	[26]
c.367C>T	p.(Gln123*)	Nonsense	Corneal haze, elevated IOP with normal cup to disc ratio	4 members (3 affected, 1 unaffected with variant), 3 generations; secondary glaucoma, phthisis bulbi, total cupping	[14]
c.437_453del17	p.(Pro146Alafs*85)	Frameshift	Glaucoma, posterior embryotoxon, iris hypoplasia, early-onset glaucoma, hearing loss, ocular hypertelorism, telecanthus, megalocornea	2 members, 2 generations; posterior embryotoxon, iris hypoplasia, corectopia, early-onset severe glaucoma, atrial septal defect, aortic stenosis, pulmonary stenosis, hearing loss, ocular hypertelorism, telecanthus	[27]
c.456G>A	p.(Trp152*)	Nonsense	Childhood-onset glaucoma with normal IOP and visual acuity, posterior embryotoxon, peripheral iridocorneal adherences, iris hypoplasia, short stature, maxillary hypoplasia, dental abnormalities	6 members, 3 generations; developmental glaucoma, posterior embryotoxon, peripheral iridocorneal adhesion, iris hypoplasia, short stature, maxillary hypoplasia, dental abnormalities,	[6]
c.477C>G	p.(Tyr159*)	Nonsense	Abnormal eye development (unspecified), leukoencephalopathy, global developmental delay, hyperreflexia	3 members, 2 generations; abnormal eye development (unspecified), leukoencephalopathy	[28]
c.609delC	p.(Ala204Argfs*111)	Frameshift	Corneal vascularization, thickened cornea (bilateral corneal opacity with peripheral corneal vascularization), corneal perforation, iris hypoplasia, posterior embryotoxon, iris heterochromia, whole globe abnormalities (unspecified), lens extrusion, persistent hyperplastic primary vitreous, retinal detachment, anterior segment developmental abnormalities, elevated IOP, mostly absent Bowman layer, absent Descemet membrane, absent endothelium, central corneal cyst	3 members, 2 generations; iris hypoplasia, posterior embryotoxon, iris heterochromia, peripheral iridocorneal adhesions, down-slanting palpebral fissures, ocular hypertelorism, malar hypoplasia, mild retrognathia, unusual dentition	[29]
c.1193_1196dupGCAA	p.(Met400Serfs*129)	Frameshift	Congenital glaucoma, microphthalmia, iris anomalies, pupil anomalies, dysmorphic facial features, bilateral club foot, mild cognitive impairment, congenital deafness	2 members, 2 generations; non-congenital glaucoma, hearing loss, club foot	[30]
c.1265C>A	p.(Ser422*)	Nonsense	Posterior embryotoxon, corectopia, megalocornea, ocular hypertension, normal systemic features	2 members, 1 generation; glaucoma, no other ocular features, normal systemic features	[31]

# Note: All nonsense truncating alleles have zero minor allele frequency in the gnomAD database. 566 total amino acids in FOXC1. IOP (intraocular pressure); fs—frameshift.

## Data Availability

Planned submission to ClinVar.
